# Chrysin Administration Protects against Oxidative Damage in Varicocele-Induced Adult Rats

**DOI:** 10.1155/2017/2172981

**Published:** 2017-12-19

**Authors:** Gabriela Missassi, Cibele dos Santos Borges, Josiane de Lima Rosa, Patrícia Villela e Silva, Airton da Cunha Martins, Fernando Barbosa, Wilma De Grava Kempinas

**Affiliations:** ^1^Department of Morphology, Institute of Biosciences, São Paulo State University (UNESP), Botucatu, SP, Brazil; ^2^Graduate Program in Cell and Structural Biology, University of Campinas (UNICAMP), Campinas, SP, Brazil; ^3^Department of Clinical Analyses, Toxicology and Food Sciences, School of Pharmaceutical Sciences of Ribeirão Preto, University of São Paulo, Ribeirão Preto, SP, Brazil

## Abstract

Oxidative stress is known as the leading factor responsible for varicocele-related infertility and for that reason, many antioxidant therapies have been proposed. Considering that, we evaluated the reproductive outcomes and fertility of varicocelized rats and the impact of chrysin within these parameters. The animals were allocated into three groups: sham (control), varicocele treated via gavage with 50 mg/kg/day of chrysin (V1), or vehicle (V2) for 56 days. Chrysin treatment prevented oxidative damage resulting from varicocele by decreasing testicular concentrations of malondialdehyde and sperm DNA fragmentation. It also improved histological aspect of the testis and maintained morphometric parameters similar to the sham group. Furthermore, there were no differences in body and reproductive organ weights, histopathological analysis of epididymis, sperm counts and morphology, testosterone levels, sexual behavior, and fertility parameters among experimental groups. Our results reinforce the idea that injuries provoked by experimental varicocele are related, at least in part, to oxidative stress. Moreover, varicocele showed bilateral deleterious effects without interfering with fertility. Chrysin administration significantly ameliorated sperm parameters, protecting the reproductive system against varicocele damages. For that reason, chrysin might be an alternative adjuvant therapy to improve sperm quality in men presenting this condition.

## 1. Introduction

Infertility is one of the main public health problems, and in more than 40% of these cases, male disorders are involved. In this context, varicocele is considered the leading cause of infertility once it is present in approximately 15% of the male population, 35% of men with primary infertility and 70–80% with secondary infertility [1, 2]. In addition, varicocele is a bilateral [3], progressive [4], and hereditary condition [5] characterized by abnormal dilatation and tortuosity of the pampiniform plexus.

Even though varicocele is known and studied for many years, the physiopathological mechanisms underlying the varicocele have not yet been fully elucidated. However, some theories that include venous stasis leading to hypoxia and testicular hyperthermia, reflux of toxic adrenal and renal metabolites, and hormonal dysfunction and increased oxidative stress (OS) have been proposed [6].

Since men with varicocele present high levels of ROS, nitric oxide (NO), and lipid peroxidation products like malondialdehyde (MDA), as well as decrease in antioxidant defenses, it is believed that OS is a key element in the varicocele-related infertility [7].

Usually, men are advised to submit to a surgical procedure known as varicocelectomy, which is considered efficient once it improves seminal and hormonal parameters [8–11]. However, the effects on fertility potential are controversial due to lack of pre- and postoperative data, adequate methodology, and criteria applied in the studies [12]. For that reason and because they offer lower risks than the surgery, several works were exploring the benefits of antioxidant adjuvant therapy [13–15]. The protective activity against oxidative stress damages and the improvement of sperm parameters make these compounds a considerable solution.

Chrysin, a flavonoid found in honey, propolis, and plant extracts, is a potent antioxidant that has been widely studied due to its mechanism of action that prevents conversion of testosterone to estrogen through inhibition of enzyme CYP19 [16, 17]. This compound is commonly used by bodybuilding athletes for being a testosterone booster, but it is also known for its antineoplastic [18], anti-inflammatory [19, 20], antihypertensive [21], anti-aging [22] activities. Several studies using animal models reported that chrysin improved sperm parameters [23, 24], increased testosterone levels [25], and prevented the oxidative damage caused by exposure to toxic chemicals [26, 27]. Thus, because this flavonoid has a positive impact on sperm quality and a protective activity against the damage caused by OS, the aim of our work was to evaluate reproductive and fertility outcomes of varicocele and the impact of chrysin in these parameters after 56 days of treatment.

## 2. Material and Methods

### 2.1. Animals

*Wistar* male (78–80 days old, weighing 300–400 g) and female rats (85 days old, weighing 200–315 g) were supplied by the Central Biotherium of São Paulo State University (UNESP), Botucatu, and maintained under controlled environment with 23°C, 12/12 h light-dark cycle with free access to food and tap water. The experimental procedures were approved by the local Ethics Committee for the Use of Experimental Animals of the University of São Paulo State (protocol number 772-CEUA) and are in accordance with the Guide for the Care and Use of Laboratory Animals (National Institutes of Health).

### 2.2. Experimental Groups and Treatment

In all experiments, male rats were allocated into three experimental groups: sham-operate (sham; *n* = 5–10) and varicocele groups received daily oral gavage dose of 50 mg/kg/day of chrysin (V1; *n* = 7-8) diluted in vehicle (corn oil and DMSO) or only vehicle solution (V2; *n* = 7–10) for 56 days. The treatment period was chosen based on the duration of spermatogenesis in rats, which is approximately 56 days, since it requires 4.5 cycles within 13 days each, of the seminiferous epithelium in order for the spermatogonia to become spermatozoa [28]. Chrysin (5,7-dihydroxyflavone) and dimethyl sulphoxide (DMSO) were purchased from Sigma-Aldrich, St. Louis MO, USA, and the dosage used was based in previous reports that 50 mg/kg/day of chrysin had a positive impact in sperm quality [23, 24] and a protective role against oxidative stress damages [19, 29, 30].

### 2.3. Surgical Procedure

Experimental left varicocele was induced on postnatal day (PND) 78–80 by partial occlusion of the left renal vein using the method described by Turner [31] with modifications. The animals were anesthetized with an intramuscular injection of ketamine 100 mg/kg and xylazine 5 mg/kg. The upper left abdominal quadrant was accessed through a midline laparotomy incision. The abdominal content was displaced to the right antimer, and the insertion of the left internal spermatic vein into the left renal vein was identified. The fat and connective tissue surrounding the renal vein was dissected, creating a tunnel underneath the vein and in a medial position to the insertion of the left spermatic vein. A 4-0 silk suture was placed around the renal vein and tied over a 0.80 mm needle. The needle was then removed, allowing the vein to expand to the limit of the ligation. Further, all abnormal collateral vessels were fully ligated once they would provide an effluent rout and release the pressure in the left internal spermatic vein. This procedure resulted in an increase in the intravenous pressure, which was transmitted to the left spermatic vein to promote varicocele development. Then, the abdominal content was replaced and the midline incision was closed in two layers with 4-0 silk suture. The animals from the sham group underwent the same procedure, but the partial ligation of the renal vein was not performed. After one week of recovery, the animals either were treated daily with chrysin/vehicle (V1/V2) or did not received any treatment (sham).

#### 2.3.1. Experiment 1. Assessment of Body and Organ Weights and Histological and Morphometric Analysis

In this experiment, Twenty-eight adult male rats (sham *n* = 10; V1 *n* = 8; V2 *n* = 10) were euthanized by decapitation at the 57th day of treatment in order to assess body and reproductive organ weights, to evaluate the histological aspect of testis and epididymis, and to perform the morphometric analysis of the testis.

### 2.4. Body and Reproductive Organ Weights

The animals were weighed daily and at the end of the treatment the testes, epididymides, prostate, and seminal vesicle were removed and had their weights recorded.

### 2.5. Histological Analysis

Testes and epididymides after weighted were fixed in Bouin's solution (25% formaldehyde, 75% saturated solution of picric acid, and 5% glacial acetic acid) for 24 h. The organs were immersed in paraffin wax and sectioned in 5 *μ*m. These sections (cross-section for testis and longitudinal sections for epididymis) were stained with hematoxylin and eosin (HE) and examined under light microscopy. Testicular evaluation was conducted by analyzing a hundred seminiferous tubules per animal, randomly chosen, and classified as normal (organized germ cell layers in the seminiferous epithelium) or abnormal (presence of acidophilic and multinucleated cells, retained spermatids, degeneration of a specific cell type, and vacuolization of the epithelium or exfoliation of cells in the lumen). The testicular interstitium was also evaluated but in a qualitative manner. Regarding the epididymis, the histological examination was qualitative. The entire longitudinal section was evaluated for the structure and aspect of epithelium, lumen, and interstitium.

### 2.6. Morphometric Analysis of Seminiferous Tubules

The diameter of the seminiferous tubules and height of germinal epithelium (stage IX of spermatogenesis) were evaluated in 10 sections of seminiferous tubules per animal.

#### 2.6.1. Experiment 2. Testosterone Levels, Sperm Parameters, and Comet Assay

Following the same experimental protocol as the previous step, twenty-four adult male rats (*n* = 8 per group) were euthanized and had their blood collected for hormonal measurements, the testis was excised for sperm count and epididymal portions were used for evaluation of sperm motility, morphology, count, and comet assay.

### 2.7. Testosterone Levels

Blood collection was performed between 8:00 and 12:00 AM. Serum was obtained by centrifugation of the samples (2500 rpm, for 20 min at 4°C) and subsequently was stored at −20°C. The concentration of testosterone (serum and intratesticular) was accessed using ImmuChem™ Double Antibody Testosterone 125I RIA Kit, and intra-assay variability for this hormone was 4.1%.

### 2.8. Sperm Motility

The epididymis was placed in a small petri dish containing 1 mL of modified human tubal fluid (HTF) medium (Irvine Scientific) prewarmed to 34°C. The proximal cauda was perforated and sperm suspension was transferred to a Makler chamber maintained at 34°C. Through a phase-contrast microscope at 400x magnification, one hundred sperm were classified as mobile and immobile.

### 2.9. Sperm Morphology

For sperm morphology analysis, 100 *μ*L of sperm solution (the same used for motility assay) was aliquoted and added to 900 *μ*L of 10% formol saline. Then, smears were prepared on histological slides, which were left to air dry for 40 minutes and observed under a phase-contrast microscope at 400x magnification. A total of 200 spermatozoa per animal were evaluated and abnormalities were classified in two main categories: head abnormalities (without characteristic curvature, pin head, and isolated head) and tail abnormalities (rolled, broken, and bended) [32, 33].

### 2.10. Sperm DNA Fragmentation (Comet Assay)

Sperm DNA fragmentation was evaluated by the comet assay as previously described [34] with the following modifications: frozen remaining sperm solution from the epididymis cauda was thawed and diluted in low melting point agarose (0.5% at 37°C; LGC Laboratories, Sao Paulo, Brazil), at a concentration of 10^6^ cells/mL. An aliquot (75 *μ*L) of the solution was placed on precoated slides with 1% normal melting point agarose (LGC Laboratories, Middlesex, UK). The slide was first immersed in lysis buffer (100 mM Na2-EDTA, 10 mM Tris-HCl, 2,5 M NaCl, and pH 11.0) containing 40 mM DTT and 2% Triton X-100 for 1 h at 4°C and subsequently in lysis buffer containing proteinase K (0.1 mg/mL), for 2.5 hrs at 37°C. The DNA was fractionated by alkaline electrophoresis (300 mM NaOH, 1 mM Na2 EDTA, and pH 13.0) at 3 V/cm and 270 mAmps for 45 mins. Finally, the slides were washed with water, fixed in absolute ethanol, and air-dried and stored in the dark until analysis. For microscopy, the slides were stained with SYBR® Gold (1 : 10,000; Invitrogen, Waltham, MA) and analyzed using the Comet Assay IV software (Comet Assay IV, Perceptive Instruments, Wiltshire, UK). A total of 50 cells were analyzed per slide (2 slides/animal) and DNA damage was measured by analyzing the tail intensity (% of migrated DNA).

### 2.11. Sperm Count

After collecting the testis from both sides, these organs were decapsulated and then frozen at −20°C until the homogenization previously described by [35], with adaptations [36, 37]. The remaining parenchyma was thawed over ice and homogenized in 5 mL of NaCl 0.9% containing Triton X-100 0.5% followed by sonication for 30 seconds. After a 10-fold dilution, an aliquot was transferred to Newbauer's chamber (4 fields per animal) and mature spermatids (stage 19 of the spermatogenic cycle) were counted. Daily sperm production (DSP) was calculated dividing the number of mature spermatids by 6.1, which is the number of days that these spermatids are present in the seminiferous epithelium. Regarding the epididymis, caput/corpus and cauda were cut in small fragments with scissors and homogenized and the counting was performed as described for the testis.

#### 2.11.1. Experiment 3. Sexual Behavior, Reproductive Performance, and Lipid Peroxidation

On the 57th day of treatment, twenty-six male rats were paired with adult females in order to evaluate sexual behavior and reproductive performance. After analyzing these parameters, the animals were euthanized by decapitation and had both testes and epididymides weighted and stored at −80°C for posterior lipid peroxidation assay.

### 2.12. Sexual Behavior and Reproductive Performance

Sexual behavior was performed on the 57th day of treatment for 4 hours in the dark phase of the cycle under red illumination. For this, 26 male rats (sham, *n* = 9; V1, *n* = 7; V2, *n* = 10) were placed in boxes of polycarbonate crystal, for 5 minutes before the introduction of one sexually receptive adult female. The behavior assay started at the time that female was placed, and the following measures were recorded for the next 40 minutes: latency of the first mount, number of mounts, latency of the first intromission, number of intromissions preceding the first ejaculation, latency of first ejaculation, and number of ejaculations [38, 39]. In case the male did not mount or intromit in the first 10 minutes after the introduction of the adult female, it was considered sexually inactive. Following the evaluation of sexual behavior, the rats (female and male) stayed in the same box for additional 4 hours, permitting a greater number of ejaculations. After this period, vaginal smears were collected for sperm detection and establishment of the day 0 of gestation (GD0). On GD21, females were weighed and killed by decapitation to enable fertility evaluation. During the laparotomy, the uterus and ovaries were collected and the number of corpora lutea, implantation sites, reabsorptions, live fetuses, and fetal weights was determined. From these results, the following endpoints were determined: pregnancy rate = number of pregnant female/number of inseminated female × 100; rate of preimplantation loss = (number of corpora lutea − number of implantations/number of corpora lutea) × 100; and rate of postimplantation loss = (number of implantations − number of live fetuses)/number of implantations × 100.

### 2.13. Lipid Peroxidation

#### 2.13.1. Homogenate Protein Determination

The testes from both sides were homogenized separately in 50 mM Tris-HCl buffer solution (pH 7.4). The protein content in the homogenate was determined by Bradford assay [40] using bovine serum albumin as a standard.

#### 2.13.2. Thiobarbituric Acid Reactive Substances (TBARS) Assay

The level of lipid peroxidation was assessed using the TBARS assay by Draper and Hadley [41]. Briefly, 200 *μ*L of the obtained homogenate was added to 500 *μ*L thiobarbituric acid (0.8%), 500 *μ*L acetic acid buffer, 200 *μ*L of SDS (8.1%), and 100 *μ*L of water. These samples were then incubated for 2 hours at 95°C and allowed to cool to room temperature. The absorbance was measured at 532 nm. All of the assays were performed in duplicate, and the amount of lipid peroxidation was expressed as nmol TBARS/g protein.

### 2.14. Statistical Analysis

Data were expressed as mean ± standard error of mean (SEM) or median and interquartile range. Parametric variables were compared by ANOVA followed by Tukey's test, nonparametric variables were compared by Kruskal-Wallis followed by Dunn's test, and *χ*^2^ test was used for categorical dependent variables. Differences were considered significant when *p* ≤ 0.05. The statistical analyses were performed by the software GraphPad Prism (version 5.0).

## 3. Results

### 3.1. Body and Organ Weights Were Not Altered by Varicocele

Final body weight and both relative and absolute reproductive organ weights were similar among experimental groups (Table 1).

### 3.2. Chrysin Improved Histological Aspect of the Testis and Maintained Morphometric Parameters Similar to the Sham Group

**Figure 1:**
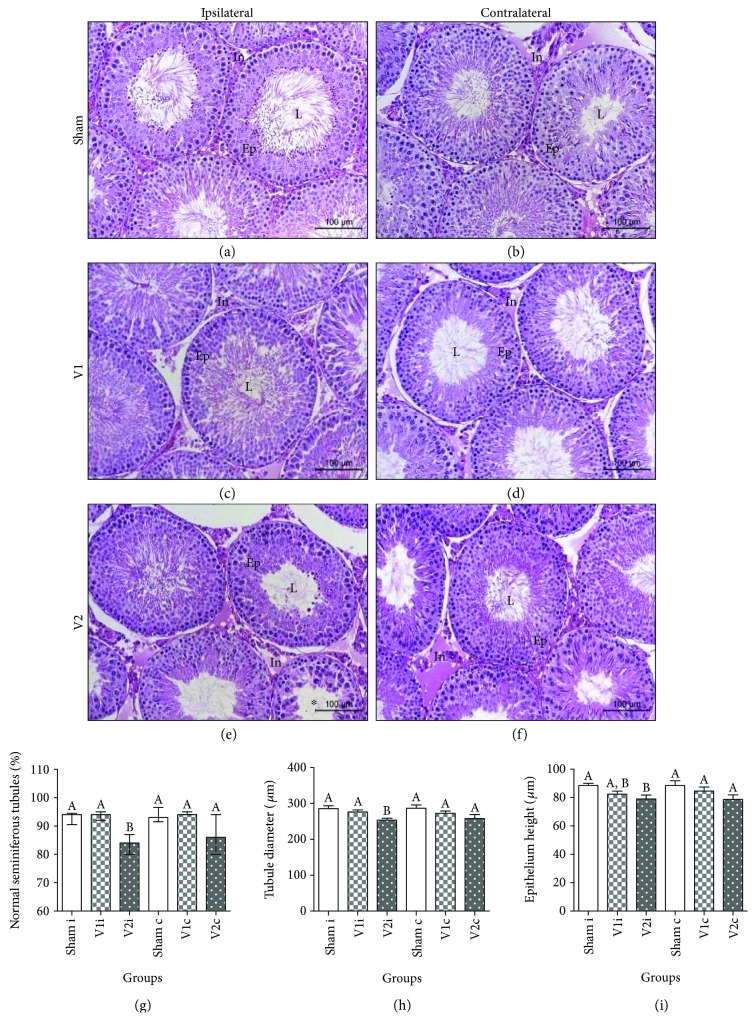
Representative histological testicular aspect in ipsilateral (a, c, e) and contralateral (b, d, f) sides. L: lumen; Ep: epithelium; In: interstitial tissue. Note, in the V2 group, there are tubules with depletion of germ cells (^∗^). Final magnification: 200x. Percentage of normal seminiferous tubules (g), tubule diameter (h), and epithelium height (i) of animals from sham, V1, and V2 groups. Values expressed as median and interquartile range. Kruskal-Wallis analysis of variance test, followed by Dunn's test (g). Values expressed as mean ± SEM, *p* ≤ 0.05. ANOVA test followed by Tukey's test (h and i). Different letters indicate *p* ≤ 0.05.

Histopathological analysis of epididymis did not reveale any alterations (data not shown) among experimental groups. However, varicocele induction significantly increased the number of seminiferous tubules with abnormalities in ipsilateral testis and the treatment with chrysin decreased this parameter to similar values to sham (Figures 1(a)–1(g)). Despite tubules from the contralateral side also presented this alteration, there were no differences compared to V1 and sham. The morphometric measurements showed that varicocele promoted a reduction in the diameter of seminiferous tubules in the ipsilateral side, and chrysin treatment maintained this parameter similar to the sham group. Additionally, the epithelial height had a significant decrease in comparison to sham. The contralateral seminiferous tubules of V2 had similar diameter and epithelium height to other groups (Figures 1(h) and 1(i)).

### 3.3. Daily Sperm Production and Epididymal Sperm Counts, Serum and Intratesticular Testosterone Levels, and Sperm Morphology Were Not Altered by Varicocele

**Figure 2:**
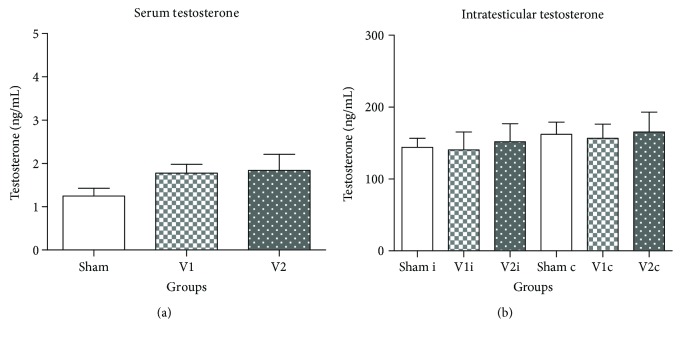
Serum testosterone levels (a) and intratesticular testosterone levels (b) in the ipsilateral (sham i, V1 i, and V2 i) and contralateral side (Sham c, V1 c, and V2 c). Values are expressed as mean ± SEM, *p* > 0.05. ANOVA followed by Tukey's test.

**Figure 3:**
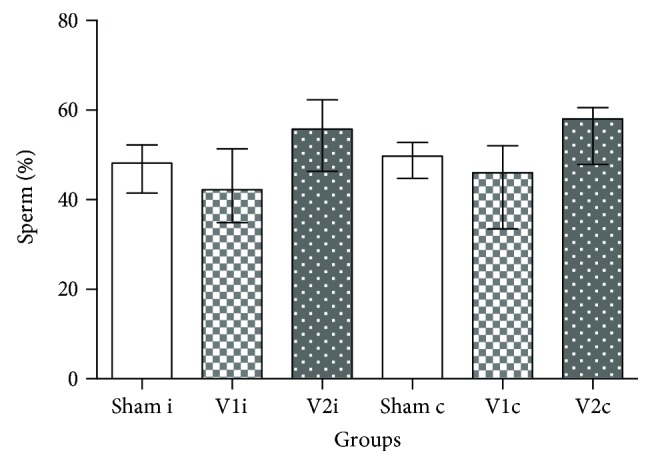
Sperm morphology. Percentage of spermatozoa with the presence of cytoplasmatic droplet in the ipsilateral (sham i, V1 i, and V2 i) and contralateral side (sham c, V1 c, and V2 c). Values are expressed as median and interquartile range. Kruskal-Wallis analysis of variance test, followed by Dunn's test (*p* > 0.05).

Sperm counts in the testis and epididymis of the V2 group (Table 2) as well as serum and intratesticular testosterone concentrations were very similar among experimental groups (Figure 2). Regarding sperm morphology, even though V2 group presented low percentages of normal sperm when compared to V1 and sham groups, the differences were not significant (Figure 3).

### 3.4. The Impairment of Sperm Motility Caused by Varicocele Was Not Significantly Improved by Chrysin Treatment

**Figure 4:**
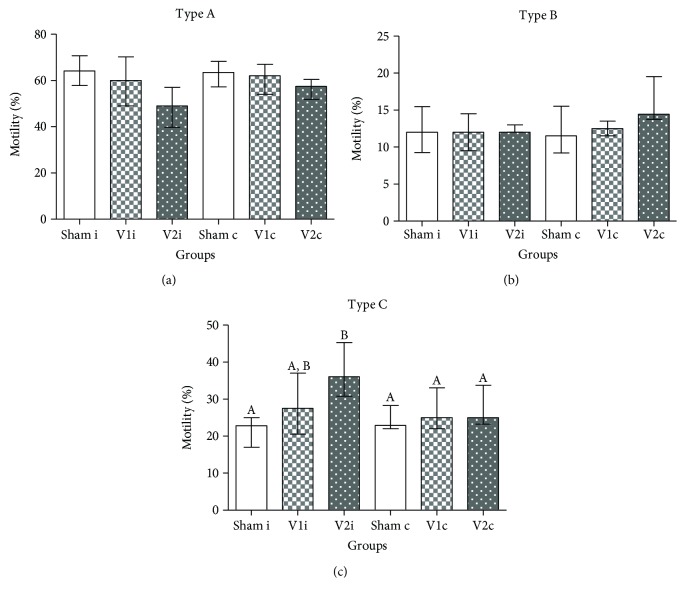
Sperm motility. Percentage of spermatozoa with type A (mobile with progression), type B (mobile without progression), and type C (immobile) in the ipsilateral (sham i, V1 i, and V2 i) and contralateral side (sham c, V1 c, and V2 c). Values expressed as median and interquartile range. Kruskal-Wallis analysis of variance test, followed by Dunn's test. Different letters indicate *p* ≤ 0.05.

V2 group presented a significant decrease in mobile sperm only when compared to the ipsilateral side of sham (*p* < 0.02), and despite the treatment with chrysin had improved this parameter, no differences were documented (Figure 4).

### 3.5. Chrysin Reduced DNA Fragmentation Caused by Varicocele

**Figure 5:**
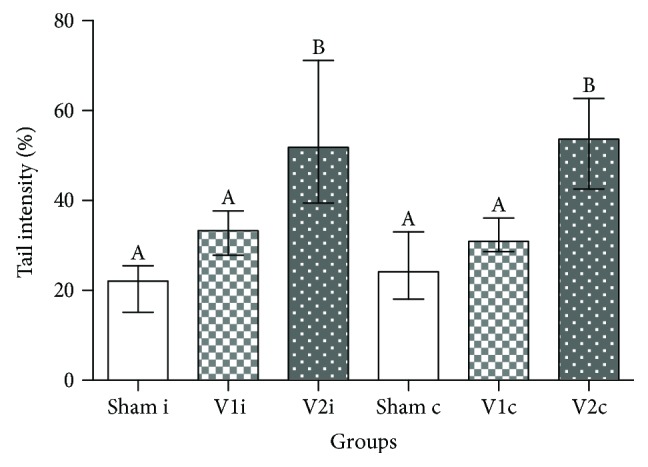
Comet Assay. Percentage of sperm DNA fragmentation through tail intensity in the ipsilateral (sham i, V1 i, and V2 i) and contralateral side (sham c, V1 c, and V2 c). Values expressed as median and interquartile range. Kruskal-Wallis analysis of variance test, followed by Dunn's test. Different letters indicate *p* ≤ 0.05.

The comet assay showed that varicocelized rats (V2) had an extremely significant increase in sperm DNA fragmentation (*p* ≤ 0.0001) in ipsilateral and contralateral sides, and the treatment with chrysin improved this parameter, decreasing the percentage of tail intensity to sham levels (Figure 5).

### 3.6. Chrysin Reduced the Levels of Testicular Malondialdehyde

**Figure 6:**
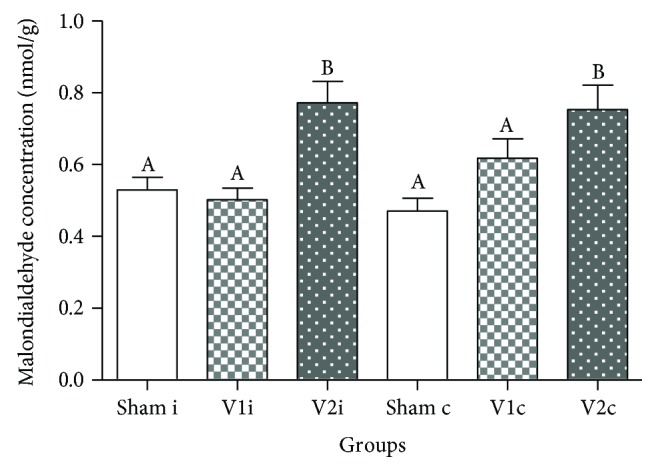
Malondialdehyde concentration in ipsilateral and contralateral testis of animals from sham, V1, and V2 groups. Values are expressed as mean ± SEM. ANOVA followed by Tukey's test was performed. Different letters indicate *p* ≤ 0.01.

The determination of testicular malondialdehyde revealed that varicocelized animals presented high concentrations of this LPO product in ipsilateral (*p* < 0.0005) and contralateral (*p* < 0.01) sides when compared to chrysin and sham groups (Figure 6).

### 3.7. Sexual Behavior or Reproductive Performance Was Not Altered by Varicocele

When sexual behavior was evaluated, there were no significant differences among experimental groups regarding the number and latency of mounts and intromissions, as well as ejaculation (Table 3). In the same manner, the reproductive performance was not significantly altered concerning pregnancy rate, pre- and postimplantation lost, weight of uterus plus fetuses, numbers of corpora lutea, implantation sites, reabsorptions, live fetuses, and fetal and dams weights (Table 4).

## 4. Discussion

 Our work is the first to report the protective effects of chrysin on reproductive disorders induced by experimental varicocele in rats. It also demonstrate the deleterious effects associated with this condition besides providing, for the first time, a more comprehensive evaluation of sexual behavior and reproductive performance in this experimental model and to evaluate intratesticular testosterone concentrations after an antioxidant treatment.

As previously reported, the principal mechanism responsible for deleterious effects of varicocele involves OS. The reactive molecules originated from OS, such as ROS, promote oxidative degradation of lipids in a process known as lipid peroxidation (LPO) [42]. Considering that sperm plasma membrane is mainly constituted by polyunsaturated fatty acids [43], these specialized cells became potential targets. Several authors have reported high levels of malondialdehyde (MDA), a LPO product in the semen [8, 44, 45] and testis [46], and showed a negative correlation with sperm parameters. In accordance with these studies [47, 48], our results revealed that varicocelized rats presented a significant bilateral increase of testicular MDA and the treatment with chrysin decreased these levels. Since several works have showed that chrysin was capable of enhancing levels of superoxide dismutase, catalase, and glutathione peroxidase in the testis [24, 49], liver [29, 50], kidney [50, 51], and colon [30], we strongly believe that this chemical probably reinforced the cell antioxidant defense preventing the increase of MDA [24, 26].

Besides compromising the integrity of the sperm plasma membrane, ROS may also interfere in cellular mechanisms through degradation of proteins. For that reason, nuclear and mitochondrial DNA of germ cells is highly susceptible to double-strand breaks [52]. Considering that the spermatozoa do not have the necessary antioxidant machinery to repair this damage, and the fact that high levels of DNA fragmentation are highly correlated with infertility [53], it is extremely important to protect this cell against ROS attack. In our experiments, chrysin treatment maintained DNA integrity similar to sham levels and varicocelized animals, in accordance with previous works [54–56], presented a significant bilateral increase in sperm DNA fragmentation. The positive impact of chrysin within this parameter might be due to its ability to increase levels of oxidative enzymes.

Moreover, another factor that is involved in the impairment of sperm quality is hypoxia. Studies have demonstrated that, along with OS [57], testis deoxygenation caused by venous stasis [58] increased the expression of pro-apoptotic proteins such as caspase [59], Bax, and Bad [60]. This combination of factors might explain the significant increase in abnormalities seen in testis histopathology of the V2 group, once the most frequent alteration observed was the depletion of germ cells. On the other hand, varicocelized rats treated with chrysin had similar percentage of normal seminiferous tubules compared to the sham group. Knowing that chrysin treatment decreased the expression of these proteins in the testis [61] and liver [26], we can conclude that this mechanism might be responsible, at least in part, for preventing cell apoptosis.

Similar to previous reports [59], another alteration verified within the testis of the V2 group was the diminution of seminiferous tubule diameter when compared to sham and V1 groups. In addition, the seminiferous epithelium in ipsilateral testis of varicocelized rats also had lower measures than the sham group. In this study, testicular temperature was not determined; however, we believe that hyperthermia, caused by venous stasis, is the probable cause of these alterations since long-term exposure to high temperatures can impair testicular morphometry [62].

Although varicocele is often associated with impaired spermatogenesis and hormonal dysfunction [63–66], our work showed that serum and intratesticular testosterone concentrations as well as daily sperm production in the testis and sperm number in epididymis were similar among experimental groups. This data contrasts with previous works that reported a decrease in serum testosterone and sperm reserves of varicocelized rats [67, 68]. Based on these observations, we can conclude that varicocele, at least in this experimental conditions, was not related to Leydig cell dysfunction which contrasts with previous reports [55]. Additionally, even though chrysin is known to prevent conversion of testosterone to estrogen, it did not influence androgen levels. We were the first to evaluate serum and intratesticular testosterone at the same time after antioxidant treatment, demonstrating that it was not altered by varicocele or chrysin treatment.

Regarding sperm motility, varicocele caused a significant decrease in mobile sperm in the ipsilateral side when compared to sham. This effect might be explained by hypoxia and by its sensitivity to oxidative stress [67]. Varicocele venous stasis decreases the supply of oxygen, which in turn increases the production of ROS. These molecules, besides interfering in the fluidity and integrity of the sperm membrane through LPO [8], can impair the synthesis of proteins involved in energy metabolism pathways, transport, and proton pumps causing energy deprivation [68]. Even though the treatment with chrysin improved this parameter, there were no differences in comparison with other groups. In the same manner and according to a study conducted by the World Health Organization [2], sperm morphology remained unaltered among experimental groups even though varicocelized rats had a lower percentage of normal sperm when compared to sham and V1.

Previous works documented, through artificial insemination [69] and natural mating [70], respectively, that pregnancy rate was significantly reduced after experimental varicocele induction. These were the only works that actually performed a fertility test on varicocelized rats, but the evidences were not sufficient to comprehend the impact that this condition has on fertility. For that reason, our work showed remarkable data regarding post- and preimplantation loss, besides giving information about dams and offspring. Furthermore, we were the first to evaluate the sexual behavior of these animals. Despite that our findings have shown that varicocele caused sperm quality impairment, there were no differences in reproductive performance and sexual behavior among experimental groups. We recognize that performing the fertility test after 56 days of chrysin treatment did not cover the whole time taken for the sperm production and transit through the epididymis and genital ducts until ejaculation. Also, natural mating may not be sensible enough to test fertility in rats. However, considering that men have lower reproductive efficiency than rats [71, 72], these results raise concern about men's reproductive health and infertility.

In conclusion, our data showed that a varicocele had a bilateral character, despite causing more severe damages in ipsilateral testis and reinforcing that OS is a key element in its pathophysiology once several testicular and sperm alterations, associated with high concentrations of MDA, were observed in varicocelized rats. It also contributed with new information regarding the sexual behavior and reproductive performance in this experimental model. Furthermore, chrysin proved to be a potent antioxidant agent, capable of protecting germ cells against the deleterious effects of varicocele, by decreasing testicular MDA concentrations and sperm DNA fragmentation and improving sperm quality. For that reason, the use of this compound as an adjuvant therapy should be considered in order to improve sperm quality of men presenting this condition.

## Figures and Tables

**Table 1:** Body and reproductive organ weights.

Parameters	Experimental groups		
	Sham (*n* = 10)	V1 (*n* = 8)	V2 (*n* = 10)
Final body weight (g)	476.2 ± 14.96	453.1 ± 17.27	472.9 ± 16.45
Testis (g)	1.83 ± 0.08	1.81 ± 0.02	1.77 ± 0.04
Testis (g/100 g)	0.38 ± 0.01	0.41 ± 0.01	0.38 ± 0.01
Epididymis (mg)	647 ± 26.74	626.1 ± 14.99	619 ± 21.08
Epididymis (mg/100 g)	135.7 ± 2.99	143.1 ± 3.12	131.5 ± 4.00
Ventral prostate (mg)	546.3 ± 38.02	612.6 ± 31.50	514.6 ± 44.68
Ventral prostate (mg/100 g)	114.1 ± 5.71	135 ± 7.11	113 ± 6.71
Seminal vesicle (g)	1.18 ± 0.12	1.25 ± 0.07	1.08 ± 0.05
Seminal vesicle (g/100 g)	0.25 ± 0.02	0.26 ± 0.02	0.23 ± 0.01

Values are expressed as mean ± SEM, *p* > 0.05. ANOVA followed by Tukey's test.

**Table 2:** Adult male rats spermatic parameters.

Parameters	Experimental groups		
	Sham (*n* = 8)	V1 (*n* = 8)	V2 (*n* = 6)
*Sperm count in the testis*			
Daily sperm production (×10^6^/testis/day)	45.79 ± 1.38	39.93 ± 0.67	42.64 ± 1.73
Relative daily sperm production (×10^6^/testis/g/day)	27.78 ± 0.63	28.21 ± 0.75	25.96 ± 1.47
*Sperm count in the epididymis*			
Caput/corpus			
Sperm number (×10^6^/organ)	197.4 ± 9.77	188.6 ± 4.42	207.2 ± 5.37
Relative sperm number (×10^6^/g/organ)	465.4 ± 24.10	500.3 ± 13.43	463 ± 28.09
Sperm transit time (days)	4.25 ± 0.27	4.62 ± 0.13	4.89 ± 0.16
Cauda			
Sperm number (×10^6^/organ)	396.0 ± 30.45	357.6 ± 18.11	382.8 ± 18.91
Relative sperm number (×10^6^/g/organ)	1458 ± 70.61	1447 ± 52.32	1451 ± 50.07
Sperm transit time (days)	6.69 ± 0.21	6.64 ± 0.25	6.96 ± 0.22

Values are expressed as mean ± SEM, *p* > 0.05. ANOVA followed by Tukey's test.

**Table 3:** Sexual behavior.

Parameters	Experimental groups		
	Sham (*n* = 9)	V1 (*n* = 7)	V2 (*n* = 10)
Latency of 1st mount (s)	66.5 ± 16.05 (9)	36.71 ± 11.47 (7)	53.50 ± 14.61 (10)
Number of mounts (*n*)	11.67 ± 2.76 (9)	15.43 ± 6.70 (7)	10 ± 1.52 (10)
Latency of 1st intromission (s)	196.7 ± 45.24 (9)	374.7 ± 127.6 (7)	328.8 ± 96.85 (9)
Number of intromissions until 1st ejaculation (*n*)	22.33 ± 4.71 (9)	24.43 ± 3.22 (7)	22.33 ± 4.49 (9)
Latency of 1st ejaculation (s)	899.1 ± 151.1 (8)	1212 ± 206.7 (5)	1050 ± 270.2 (5)
Number of ejaculations (*n*)	2.43 ± 0.43 (7)	1.33 ± 0.21 (5)	2.0 ± 0.55 (5)

Values are expressed as mean ± SEM, *p* > 0.05. ANOVA followed by Tukey's test.

**Table 4:** Fertility test after natural mating.

Parameters	Experimental groups		
	Sham (*n* = 7)	V1 (*n* = 8)	V2 (*n* = 7)
^1^Pregnancy rate (%)	66.66	100	70
^2^Preimplantation loss (%)	0.00 (0.00–12.50) (6)	8.33 (7.69–25.00) (7)	0.00 (0.00–7.69) (7)
^2^Postimplantation loss (%)	0.00 (0.00–2.27) (6)	0.00 (0.00–8.33) (7)	0.00 (0.00–9.09) (7)
^3^Body weight of dams (g)	364 ± 11.54 (7)	365 ± 15.78 (8)	379.9 ± 9.92 (7)
^3^Uterus weight with fetuses (g)	69.16 ± 6.89 (7)	74.36 ± 4.32 (8)	76.05 ± 4.2 (7)
^3^Corpora lutea number	12.57 ± 0.72 (7)	13.25 ± 0.75 (8)	12.71 ± 0.56 (7)
^3^Implantation number	12.00 ± 0.62 (7)	11.5 ± 0.63 (8)	12.29 ± 0.42 (7)
^3^Number of live fetuses	10.86 ± 1.32 (7)	11.25 ± 0.70 (8)	11.71 ± 0.64 (7)
^3^Fetus weight (g)	4.61 ± 0.20 (7)	4.71 ± 0.07 (8)	4.74 ± 0.13 (7)

^1^ *χ* ^2^ test with results expressed in percentage. ^2^Values are expressed as median and interquartile intervals (Kruskal-Wallis test followed by Dunn's test, *p* > 0.05). ^3^Values are expressed as mean ± SEM, *p* > 0.05 ANOVA followed by Tukey's test.
